# Effects of Steam Explosion-Assisted Extraction on the Structural Characteristics, Phenolic Profile, and Biological Activity of Valonea

**DOI:** 10.3390/foods14234096

**Published:** 2025-11-28

**Authors:** Zhenkai Tong, Wenjun Li, Jianxin Jiang, Chengzhang Wang

**Affiliations:** 1 State Key Laboratory of Efficient Utilization of Forest Food Resources, Institute of Chemical Industry of Forest Products, Chinese Academy of Forestry, Nanjing 210042, China; 18036262726@163.com (Z.T.);; 2 College of Materials Science and Technology, Beijing Forestry University, Beijing 100083, China; 3 Key Laboratory of Biomass Energy and Material, Nanjing 210042, China; 4 Key Laboratory of Chemical Engineering of Forest Products, National Forestry and Grassland Administration, Nanjing 210042, China; 5 International Innovation Center for Forest Chemicals and Materials, Jiangsu Co-Innovation Center for Efficient Processing and Utilization of Forest Resources, Nanjing 210042, China

**Keywords:** steam explosion, total polyphenols, valonea, antioxidant activity, anti-inflammatory activity

## Abstract

Valonea, a natural product from *Quercus variabilis*, is rich in bioactive phenolic compounds; however, its compact physical structure restricts the efficient extraction of these components, limiting its high-value applications. To address this issue, the present study examined the influence of steam explosion (SE) pretreatment on the physical structure, phenolic profile, and bioactivity of valonea and identified optimal processing parameters. Under optimal conditions (1.0 MPa and 10 min), the content of total polyphenols increased by 63.1%, reaching 553.0 mg/g extract, while gallic acid and ellagic acid concentrations increased by 380.6% and 1280.0%, respectively. Electrospray ionization mass spectrometry identified 12 major phenolic constituents, providing a compositional basis for the observed bioactivities. The extract exhibited strong antioxidant and anti-inflammatory properties, confirming that SE not only augments phenolic content but also preserves or enhances the functional quality of the extract. As an efficient and environmentally friendly pretreatment technology, SE substantially improves the bioavailability and activity of phenolics in valonea. Thus, this study offers a reliable strategy for the high-value utilization of valonea in fields such as pharmaceuticals, functional foods, and animal feed.

## 1. Introduction

*Quercus variabilis* (Chinese cork oak), a key tree species in East Asian forests, produces valonea—the protective cup that detaches from its acorn following maturity [1,2]. This agricultural byproduct is gaining attention not only for its ecological role but also for its high content of hydrolyzable tannins and other polyphenolic compounds, which underlie its remarkable potential for valorization [3]. Hydrolyzable tannins in valonea mediate a range of important bioactivities, including antibacterial, antidiarrheal, and antioxidative effects [4]. Industrially, these compounds serve as viscosity reducers in drilling fluids and effective coagulants in wastewater treatment; they are also popular as the “soul of wine” in oenology for imparting characteristic astringency and flavor to wine [5,6,7]. Despite this promising profile, valonea remains largely underutilized and is primarily used as a low-value combustion feedstock in China. This underutilization stems from a critical technological limitation: the inefficient extraction of bioactive phenolics from the dense, recalcitrant structure of valonea, which hinders its industrial-scale applications.

While numerous advanced methods exist to improve polyphenol extraction, including solid/liquid, supercritical, pressurized water, microwave-assisted, and ultrasound-assisted extraction [8], hot water extraction as the conventional technique is comparatively simple but suffers from low efficiency, high energy demand, and inferior bioactivity of the extracts [9]. These limitations, often caused by the low soluble nature of conjugated phenolic compounds in solvents, constrain the application of traditional methods [10]. SE technique exposes materials under elevated temperatures and pressures. The instantaneous release of pressure vaporizes the superheated moisture within the interstices of the raw material, resulting in a sharp volumetric expansion that disrupts the cellular structure. This structural disintegration of the tough plant material facilitates the release and dissolution of desired compounds, ultimately improving both biological activity and comprehensive utilization rates [11,12,13,14]. As a green and efficient process, SE offers distinct advantages for rigid plant materials, including high extraction yield, low cost, and minimal environmental impact. As an established pretreatment process, SE has been used to extract various bioactive compounds, including dietary fiber [4], proteins, cellulose, polysaccharides, and phenols [11,15,16,17,18,19,20]. The synergistic action of heat and pressure during SE causes more substantial alterations in the plant matrix than that achieved through individual treatments; this feature is highly beneficial for releasing phenolic compounds. For instance, SE pretreatment markedly increased the total phenolics and enhanced the antioxidant activity in extracts obtained from mung beans and pomegranate peel and promoted the dissolution of specific phenolic acids in pomegranate peel extract [21,22]. Our previous work further confirmed that optimized SE parameters (1.3 MPa and 30 s) substantially increased the total polyphenol (TP) content of *Baphicacanthus cusia* extract by 74.52% and its antioxidant activity by 38.16–109.14% [23].

This collective evidence confirms the potential of SE in disrupting lignocellulosic structures to release phenolics. While valonea presents a structurally promising candidate, the specific effects of SE on its structural modification, phenolic extraction efficiency, compositional profile, and bioactivity remain unexplored. To address this research gap, the present study is based on the hypothesis that SE synergistically uses physical and chemical forces to disrupt the recalcitrant lignocellulosic structure of valonea, thereby enhancing the release, dissolution, and bioactivity of its bound phenolics. The specific objectives were (1) to systematically characterize the structural and compositional alterations in valonea from macro to micro levels by colorimetry, scanning electron microscopy, and spectroscopy; (2) to determine the optimal SE parameters for maximizing polyphenol yield; (3) to analyze the dynamic changes in the phenolic profile; and (4) to assess the improvement in the antioxidant and anti-inflammatory capacity of the resulting extracts. Collectively, this research paves the way for the high-value exploitation of valonea resources by establishing a solid theoretical and technical foundation.

## 2. Materials and Methods

### 2.1. Reagents and Materials

Valonea samples were collected from Luodian County, Guizhou, China, in September 2024. The source trees were identified as *Q. variabilis* by our institute’s professor. Post-collection, the samples were transported through cold chain and stored at −80 °C. Gallic acid (>99%), ellagic acid (>99%), methanol (>99%), anhydrous sodium carbonate (>98%), Folin–Ciocalteu reagent (>99%), RAW 264.7 murine macrophages, Dulbecco’s Modified Eagle’s Medium (DMEM), fetal bovine serum (FBS), lipopolysaccharide (LPS), and penicillin-streptomycin solution (P/S) were obtained from Sinopharm Chemical Reagent Co., Ltd. (Shanghai, China). ABTS (>98%), DPPH (>95%), FRAP (>98%), Cell Counting Kit-8 (CCK-8) assay and enzyme-linked immunosorbent (ELISA) assay kits were purchased from Shanghai Jining Biotechnology Co., Ltd. (Shanghai, China). Other chemical reagents were of pure and analytical grade.

### 2.2. SE Pretreatment

Pretreatment of valonea was conducted using a SE reactor (Model FIBERPULP-SE-100, Parker Machinery Technology Co., Ltd., Wuhan, China). An untreated sample was rigorously analyzed as the negative control. Following exploratory studies, the SE parameters were optimized by varying the pressure (0.5–2.0 MPa) and residence time (6 and 10 min) within the reactor, which yielded eight pretreated samples, designated as SE2 to SE9. SE-pretreated valonea was recovered, air dried at 25 °C, and stored in a dryer until analysis.

### 2.3. Physicochemical Properties

#### 2.3.1. Color Measurement

The chromatic values (L*, a*, b*) of both untreated and SE-pretreated valonea were measured using a color spectrometer (CR-400, Konica Minolta, Inc., Tokyo, Japan). This approach provided an objective, quantitative evaluation of surface color changes, eliminating the bias of subjective visual assessment. All measurements were carried out under identical ambient lighting conditions following instrument calibration. The L* value corresponds to lightness, while a* and b* represent the green–magenta and blue–yellow components, respectively. The comprehensive color difference, ΔE*, was calculated as √(ΔL^2^ + Δa^2^ + Δb*^2^), where ΔL*, Δa*, and Δb* are the relative differences in lightness, red-green coloration, and yellow-blue coloration between the untreated and SE-pretreated valonea, respectively.

#### 2.3.2. Morphological Analysis

Scanning electron microscopy (SEM; Model 3400-I, Hitachi Corporation, Tokyo, Japan) was employed to characterize the surface morphology of the samples, as this technique provides high-resolution topographical information that directly reveals microstructural features such as particle shape and porosity. Due to the insulating nature of the samples, all were uniformly coated with a gold layer via sputtering to enhance surface conductivity. To circumvent this issue and prevent potential electron beam damage, all observations were conducted under identical operating conditions, including an acceleration voltage of 2 kV and a magnification of 1500×, thereby ensuring the acquisition of reliable microstructural data.

#### 2.3.3. Fourier Transform Infrared (FTIR) Spectroscopy

With the objective of identifying changes in key functional groups and molecular structures, the impact of SE pretreatment on valonea was analyzed by FTIR spectroscopy on a Nicolet iS50 spectrometer (Thermo Fisher Scientific, Waltham, MA, USA). The spectra were recorded from 4000 to 400 cm^−1^ at a resolution of 4 cm^−1^. Multiple scans were averaged for each sample to enhance the signal-to-noise ratio, with background scans periodically conducted under identical settings to eliminate environmental contributions.

#### 2.3.4. Powder X-Ray Diffraction (XRD) Analysis

To assess changes in the cellulose crystallinity of valonea induced by SE pretreatment, XRD analysis was performed using an X-ray diffractometer (Model D8 FOCUS, Bruker AXS GmbH, Karlsruhe, Germany) with Cu Kα radiation (40 kV, 15 mA). All samples were uniformly packed into the sample holder, and data were acquired using a fixed scan rate and step size across the 2θ range of 10–70° at a scan rate of 0.4°/minto ensure comparability.

### 2.4. Extraction of Phenolic Compounds from Valonea

#### 2.4.1. Extraction and Identification of Phenolic Compounds

Extracting Valoneta using the most efficient solvent extraction method. Prior to further analysis, each 20 g sample was subjected to water-based extraction at a 1:20 (g:mL) solid–liquid ratio with continuous stirring at 55 °C for 45 min. After centrifugation (3000 rpm, 10 min),he filtered (0.22 μm) supernatants were assessed for total phenolics using the Folin–Ciocalteu assay, with concentrations expressed as milligrams of gallic acid equivalent per gram (mg GAE/g dry extract). The contents of two key phenolic compounds—gallic acid and ellagic acid—were measured by high-performance liquid chromatography by using a previously reported procedure with slight modifications [13]. The analysis was performed on an Agilent 1260 Infinity II HPLC system (Agilent Technologies, Inc., Santa Clara, CA, USA) equipped with a ZORBAX Eclipse Plus C18 column (150 mm × 4.6 mm, 5 µm). Separation was achieved using a gradient mobile phase of (A) methanol and (B) water (both containing 0.1% trifluoroacetic acid) at a flow rate of 1.0 mL/min. The gradient program was set as follows: 0–3 min, 10% A; 3–25 min, 10–30% A; 25–50 min, 30–80% A; 50–55 min, 80–10% A; and 55–60 min, 10% A. The flow rate was maintained at 1.0 mL/min, with detection at 280 nm and column oven temperature at 28–32 °C.

#### 2.4.2. Kinetics of Polyphenol Extraction

To investigate the release kinetics of polyphenols and provide a theoretical basis for process optimization, samples were extracted using the aforementioned method over varying time periods (5, 10, 20, 30, 50, 70, 90, 120, and 180 min). Based on the four-parameter sigmoidal model (also known as the generalized logistic function) [24], the release kinetics of phenolic compounds from valonea under different SE conditions were investigated. The theoretical framework operates on the following specific conditions: phenolic compounds are evenly distributed in spherical particles, the diffusion coefficient is invariant, and the effects of external mass transfer are minimal. This correlation was quantitatively described by the equation Y = A_2_ + (A_1_ − A_2_)/[1 + (x/x_0_)^p^], where Y is the phenolic yield at time t (mg/g), x is the extraction time (min), A_1_ is the initial content (mg/g), A_2_ is the maximum theoretical content (mg/g), x_0_ is the half-saturation time (min), and p is an extraction rate factor.

### 2.5. Identification of Phenolic Compounds

Electrospray ionization mass spectrometry (ESI-MS) was conducted with a Q Exactive Orbitrap mass spectrometer (Thermo Fisher Scientific, Bremen, Germany) to enable the accurate identification of complex phenolic compounds. Following a 2-μL loop injection with a methanol/acetonitrile mobile phase (200 μL/min), analyses were conducted in both positive and negative full MS modes by using a heated electrospray ionization source. The mass resolution was set to 140,000 (at *m*/*z* 200), with spray voltages at 3.50 kV (positive) and 3.00 kV (negative). Additional parameters were as follows: sheath/auxiliary gas flow, 45/15 arbitrary units; capillary temperature, 250 °C; and S-lens re-imagined focus level, 50 V.

### 2.6. Determination of Antioxidant Activity

Antioxidant activity was evaluated using three widely recognized assays (ABTS, FRAP, and DPPH). The ABTS and FRAP assays were carried out using commercial kits in strict adherence to the manufacturer’s protocols. DPPH radical scavenging activity was determined according to the method of [13] with slight modifications [13]. The reaction between 0.4 mL of extract and 0.6 mL of DPPH solution was carried out in the dark (25 °C, 30 min), and the absorbance was subsequently measured at 515 nm. Results were expressed as µmol Trolox equivalents per gram (µmol TE/g).

### 2.7. Anti-Inflammatory Activity Assay

The anti-inflammatory potential of valonea polyphenolic extract was examined in LPS-induced RAW 264.7 macrophages. Prior to this assessment, the cells were routinely maintained in DMEM supplemented with 10% FBS and 1% P/S at 37 °C in a 5% CO_2_ atmosphere and passaged every 2 days. For the experiment, cells were plated in 24-well plates at 2 × 10^4^ cells/mL and allowed to adhere for 24 h. The concentration range of the extract (12.5–400 μg/mL) was selected based on a preliminary cytotoxicity assay. Cells were pretreated with the extract at the indicated concentrations for 1 h, followed by co-incubation with 1 µg/mL of LPS for 24 h to establish an inflammatory model. Cell viability was confirmed using the CCK-8 assay to ensure no cytotoxicity at all tested concentrations. Nitric oxide (NO) production was measured in the culture supernatant using the Griess method. Additionally, the secretion levels of the key proinflammatory cytokines tumor necrosis factor (TNF)-α, interleukin (IL)-6, and IL-1β were determined by ELISA at three non-cytotoxic concentrations (100, 200, and 400 μg/mL), together with the control and LPS groups.

### 2.8. Statistical Analysis

All data are presented as the mean ± standard deviation (SD) from at least three independent experimental replicates. The normality and homogeneity of variances of the datasets were confirmed by the Shapiro–Wilk and Levene’s tests, respectively. Statistical comparisons among multiple groups were analyzed by one-way analysis of variance (ANOVA), followed by Tukey’s post hoc test for specific pairwise comparisons. Employing IBM SPSS Statistics software (v19.0; IBM Corp., Armonk, NY, USA), statistical significance was defined at *p* < 0.05.

## 3. Results and Discussion

### 3.1. Valonea Appearance and Microstructure

**Figure 1:**
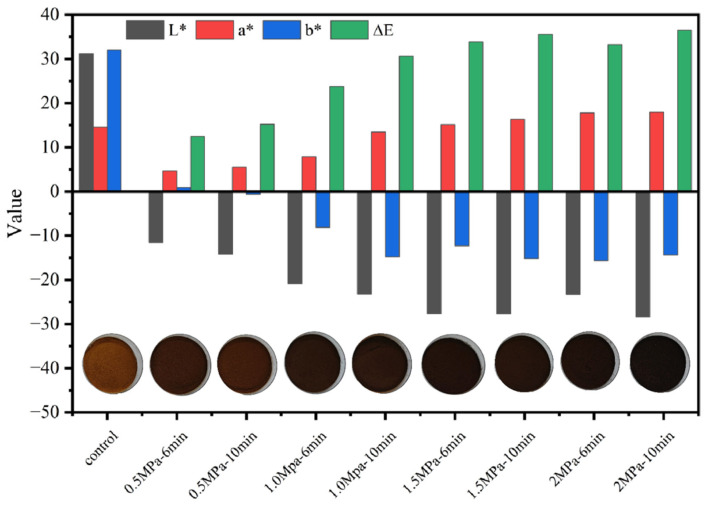
Color comparison results of valonea under different SE conditions (corresponding to L*, a*, and b* values).

The visual appearance of valonea after SE pretreatment with varying pressure (0.5–2.0 MPa) and residence time (6 and 10 min) in a SE reactor is illustrated in Figure 1. Statistical analysis revealed that the total color difference (ΔE*) increased markedly (*p* < 0.05) with the severity of SE, confirming substantial visual alteration.

This color evolution was characterized by a systematic and significant decrease in L* value (*p* < 0.001), indicating pronounced sample darkening, coupled with a significant increase in a* values (*p* < 0.001) and a significant decrease in b* values (*p* < 0.01), which collectively indicated a distinct color shift toward reddish-brown hues. The observed pattern of darkening and red shift is a documented phenomenon in lignocellulosic biomass following exposure to thermomechanical treatments. Although not directly quantifying reaction intermediates, the pronounced color shifts offer indirect evidence of SE-induced chemical alterations. The underlying mechanism for this browning is likely multifaceted. A primary contributing factor is thought to be the Maillard reaction. This is hypothesized on the well-established understanding that SE conditions readily hydrolyze hemicellulose, releasing soluble sugars (e.g., xylose and glucose), which can then react with amino compounds at high temperatures to form brown, polymeric melanoidins [25]. Concurrently, the thermal degradation and condensation of lignin, which generates its own suite of chromophoric structures, undoubtedly contributes to the darkening effect [26]. The progressive nature of the color change with increasing pressure and residence time highlights the amplification of these underlying chemical pathways. Thus, the observed colorimetry robustly indicates that SE pretreatment alters the chemical profile of valonea.

**Figure 2:**
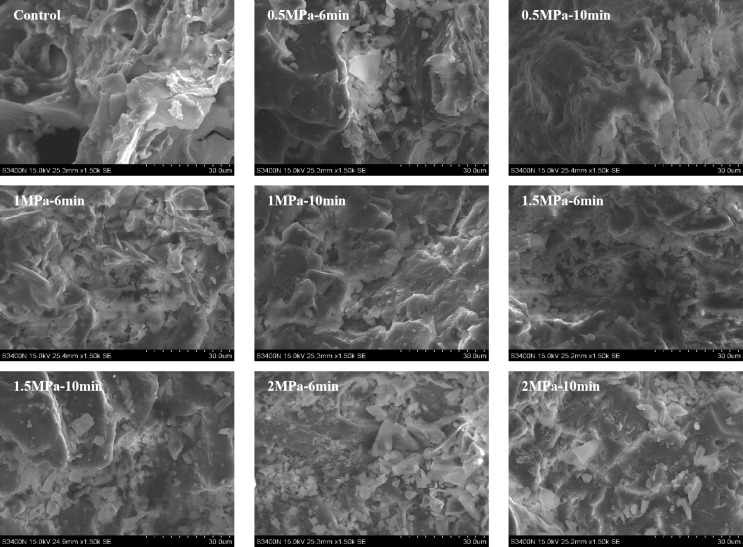
SEM images of valonea following SE pretreatment under different conditions (magnification, 1500×).

SEM images of valonea following SE pretreatment under different conditions are shown in Figure 2. The untreated valonea had a smooth surface with clear and neatly arranged pores; however, after SE pretreatment, the microstructure of valonea clearly changed. Valonea pellets were broken into smaller fragments; moreover, with the increase in SE severity, the valonea fragmentation degree further increased, with a dry and rough appearance and new cracks and holes on the surface. Under 0.5 MPa pressure, the blasted raw material showed apparent bending, surface shrinkage and grooves, and a small amount of fragmentation; in contrast, at 1.0 MPa pressure and 10 min residence time, the degree of fragmentation had increased as compared to that at 0.5 MPa and 10 min, with curling and folding of fibers and the appearance of deeper cracks. At 1.5 MPa pressure, the fiber structure was damaged, and more fragments appeared. A further increase in pressure to 2.0 MPa severely damaged the fiber structure of valonea, leading to curved and deep cracks along with the ejection of numerous minute fragments. These findings demonstrated that the extent of structural damage to valonea was positively correlated with the intensity of SE pretreatment. These structural changes can be attributed to a two-stage mechanism: first, high-temperature, high-pressure steam penetrated the cell walls through microporous structures, leading to explosion and fragmentation, followed by the instantaneous release of pressure, which resulted in expansion and flash evaporation, collectively disrupting the architecture of the material [27,28]. Properly controlled SE-induced structural modifications in valonea are conducive to improving the solubility and accessibility of its bioactive components, thereby influencing the leaching behavior and bioactivities of phenolic compounds [29].

### 3.2. FTIR Spectroscopy of Valonea

**Figure 3:**
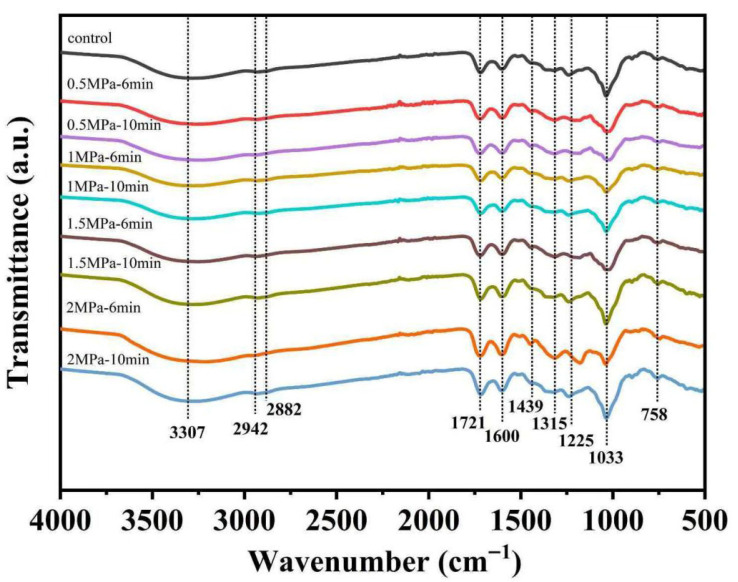
FTIR spectra of valonea following SE pretreatment under different conditions.

As shown in Figure 3, the FTIR spectra were used to determine the molecular-level structural modifications in valonea induced by SE pretreatment. A comparative analysis of the spectra between the raw and SE-treated samples revealed significant changes in key functional groups, primarily indicative of the depolymerization and cleavage of lignocellulosic components.

The interpretation of the major spectral changes is as follows. The broadening and increased intensity of the O-H stretching band (~3307 cm^−1^) in SE-treated samples suggest a disruption of the native hydrogen-bonding network within cellulose and hemicellulose. This is attributed to steam penetration and violent decompression, which expose more free hydroxyl groups and enhance cell wall accessibility. The intensity of the carbonyl (C=O) stretching band at ~1721 cm^−1^, primarily attributed to acetyl esters in hemicelluloses, was observed to decreased after SE treatment. This spectroscopic result is direct evidence for the deacetylation and depolymerization of hemicelluloses. High-temperature steam hydrolyzes ester linkages, releasing acetic acid that further catalyzes glycosidic bond hydrolysis [30].

The aromatic and ether linkage region (1600 cm^−1^ and 1225 cm^−1^): The band at ~1600 cm^−1^, assigned to aromatic C=C skeletal vibrations in lignin, showed a relative increase in intensity. This is not due to an increase in lignin content, but rather a concentration effect resulting from the preferential removal of polysaccharides (hemicellulose). Additionally, it may indicate lignin repolymerization or condensation reactions, which are known to occur under severe thermochemical treatments. Concurrently, the band at ~1225 cm^−1^, associated with C-O-C stretching in aryl-alkyl ether linkages (e.g., the β-O-4 bonds in lignin), was notably diminished. This provides direct evidence for the cleavage of the vital ether bonds within the lignin polymer and the lignin-carbohydrate complexes. The fragmentation of lignin is a key factor in breaking the rigid cell wall structure. The C-O stretching vibration at ~1033 cm^−1^, characteristic of pyranose rings in cellulose and hemicellulose, became broader and less resolved, indicating structural disorder and amorphization of cellulose and hemicellulose, consistent with their partial degradation [31].

In summary, the FTIR analysis conclusively demonstrates that SE pretreatment induces profound chemical changes in valonea structure. The key mechanisms include (1) the hydrolysis of hemicellulose, (2) the cleavage of lignin ether bonds, and (3) the disruption of the hydrogen-bonding network in carbohydrates. These changes collectively dismantle the dense lignocellulosic matrix, forming micropores and pathways that enhance the release of bound phenolic compounds, thereby validating SE as an effective pretreatment.

### 3.3. XRD Analysis of Valonea

**Figure 4:**
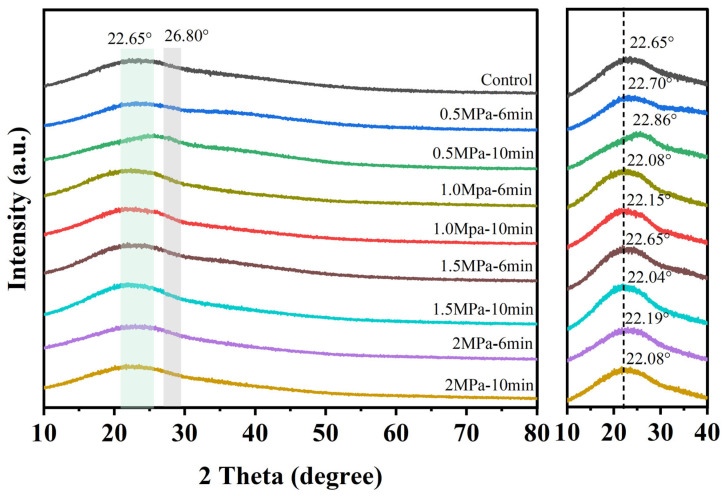
XRD patterns of valonea following SE pretreatment under different conditions.

Modifications to the cellulose crystallinity of valonea induced by SE pretreatment were evaluated by XRD (Figure 4). The XRD pattern of the untreated sample exhibited a distinct diffraction peak at 22.65°, characteristic of cellulose I crystalline structure. In contrast, SE-pretreated samples exhibited progressive peak weakening and broadening with increasing pretreatment severity, with the highest reduction observed under the most severe condition (2 MPa and 10 min), while maintaining cellulose I form.

These changes reflect reduced crystallinity and enhanced structural disorder, attributable to the synergistic thermal–mechanical–chemical effects of SE. The rapid pressure release generated substantial shear forces that disrupted the hydrogen-bonding network within the crystalline domains, leading to cellulose chain displacement and increased amorphous regions [32,33]. This microstructural disruption facilitated cell wall breakdown and microfibril separation. Notably, the peak intensity at 26.8° displayed a non-monotonic response to pressure increase, suggesting an initial pressure-induced alignment followed by structural loosening under severe conditions. This complex structural evolution, accompanied by increased specific surface area, ultimately resulted in decreased crystallinity and peak broadening [34].

These structural modifications directly enhanced the efficiency of phenolic compound extraction. The reduced crystallinity and increased amorphous regions improved solvent accessibility and mass transfer, facilitating the release of phenolics otherwise trapped in the crystalline cellulose matrix. This molecular-level insight into SE-induced structural changes explains the significantly improved extraction yields, demonstrating how the disruption of cellulose crystalline structure can enhance the application of valonea in biorefinery.

### 3.4. Analysis of the Polyphenol Composition of Valonea

**Figure 5:**
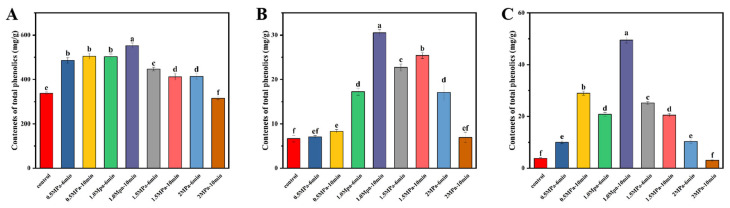
(**A**) TP, (**B**) gallic acid content, and (**C**) ellagic acid content of valonea subjected to SE pretreatment under different conditions. Bars labeled with different letters are significantly different (*p* < 0.05) as determined by one-way ANOVA followed by Tukey’s post hoc test.

The TP content of valonea, along with the concentrations of gallic acid and ellagic acid, was quantified before and after SE pretreatment. As illustrated in Figure 5, SE pretreatment markedly increased the TP content in the extract, along with an increase in gallic acid and ellagic acid contents. After treatment, with the increase in ultrasonic intensity, the TP content first increased and then decreased. Compared to the pretreatment level, the TP level post-treatment increased by approximately 63.1% (from 339.0 to 553.0 mg/g). Under the optimal SE conditions of 1.0 MPa and 10 min, the highest extracted polyphenol content reached 553.0 mg/g. Similarly, gallic acid and ellagic acid contents rose markedly, reaching 29.8 mg/g and 48.3 mg/g, the elevated values represent a substantial increase of approximately 380.6% and 1280.0%, respectively.

The observed enhancement in TP content stems fundamentally from the synergistic physicochemical actions of SE pretreatment. The rapid pressure release disrupts the lignocellulosic matrix, while high temperature and pressure hydrolyze structural polymers. This dual action disrupts the covalent bonds (particularly ester and ether linkages) between cell wall components (cellulose, hemicellulose, and lignin) and bound phenolic compounds. This mechanistic framework explains the synchronized release of gallic acid, often bound as hydrolyzable tannins or esters of cell wall polysaccharides, and ellagic acid, typically present in glycosylated forms or as ellagitannin polymers. The simultaneous increase in these specific phenolic acids confirms that SE effectively targets different binding mechanisms within the plant matrix. However, the release process exhibits a distinct threshold. Beyond optimal intensity, the enhanced accessibility and exposure render phenolic structures vulnerable to thermal degradation and oxidative reactions. The observed decline in extractable polyphenols under severe conditions (e.g., very high pressure or extended duration) aligns with reported phenomena in other plant systems and can be attributed to the decomposition of labile phenolic structures, polymerization reactions, and potential Maillard reaction with co-extracted sugars [14,21,23,25,35]. Thus, the optimal condition represents a balance between structural disruption for phenolic release and avoidance of destructive side reactions.

Therefore, the optimal SE condition of 1.0 MPa pressure for 10 min represents a processing window that maximizes the breakdown of the lignocellulosic barrier for phenolic release while minimizing destructive side reactions. This understanding advances the fundamental rationale for using SE in biorefinery applications, positioning it not only as a physical pretreatment but also as a targeted process for mobilizing bound phytochemicals through controlled structural fragmentation.

### 3.5. Kinetics of Extraction of Phenolic Compounds from Valonea

**Figure 6:**
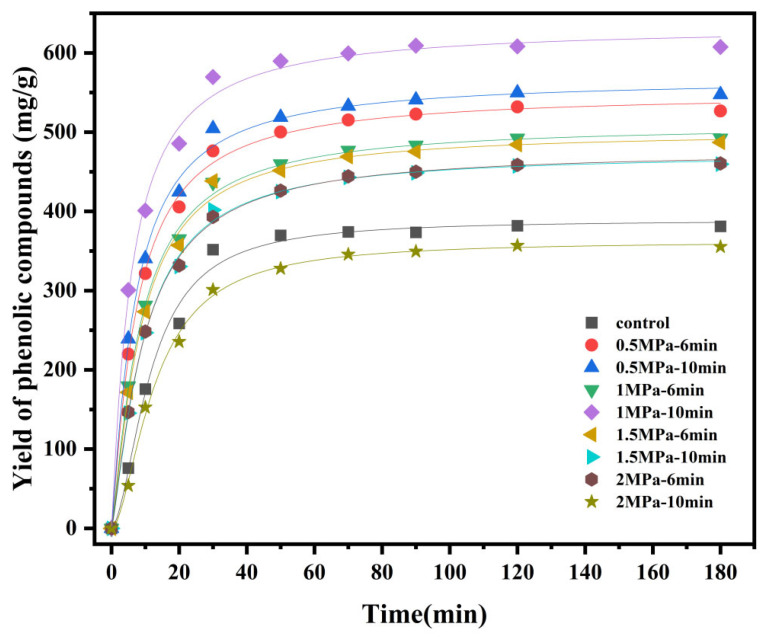
Extraction kinetics of TP from valonea subjected to SE pretreatment under different conditions.

We applied a four-parameter generalized logistic model to analyze the polyphenol extraction kinetics from SE-pretreated valonea. As shown in Figure 6 and Table 1, the coefficient of determination (R^2^) for all fitted curves exceeded 0.99, indicating that the model provides an extremely accurate description of the dynamic polyphenol extraction process.

Parameter A_2_, the theoretical maximum yield, is a key indicator of extraction efficiency. Compared to the untreated control (A_2_ = 388.95 mg/g), most SE pretreatments significantly increased (*p* < 0.05) the maximum yield, demonstrating their effectiveness in enhancing polyphenol accessibility. The optimal pretreatment of 1 MPa for 10 min yielded the highest A_2_ value of 633.62 mg/g, an increase of approximately 63% over the control. Notably, the improvement in extraction efficiency was not positively correlated with treatment intensity. Under very high treatment pressure or very long treatment duration, such as 1.5 MPa for 10 min as well as 2 MPa for 6 and 10 min each, the A_2_ values were lower than that for moderate intensity treatment (1 MPa) or even lower than that of the control group (2 MPa, 10 min; A_2_ = 362.19 mg/g). This indicates an optimal pretreatment window, beyond which excessively severe conditions may degrade thermolabile polyphenols or promote their irreversible binding with other cellular components like proteins and sugars, thereby reducing extractable content.

Beyond the final yield, the extraction kinetics were also enhanced. A decrease in the parameter x_0_ directly reflects an accelerated extraction rate. The control group required 11.54 min to reach half of the maximum yield, whereas all effective SE pretreatments reduced the x_0_ value. The optimal condition (1 MPa, 10 min) halved the x_0_ to 5.77 min, indicating a doubling of the extraction rate. Similarly, the conditions of 0.5 MPa for 6 min and 10 min each also exhibited fast extraction kinetics (x_0_ ≈ 7.30 and 6.84 min, respectively). This phenomenon indicates that SE disrupted the plant cell wall structure, impairing the encapsulation and barrier effect of lignocellulose on polyphenols, thus allowing water to penetrate more rapidly into the cell interior; This structural disruption allows solvent to penetrate faster and polyphenols to diffuse out more efficiently, shortening the time to reach equilibrium. Changes in the *p*-value reveal a shift in the extraction kinetic pattern. The control group had a relatively high *p*-value (1.77), indicating a steeper extraction curve, where the extraction rate increased rapidly after a relatively slow initial phase until equilibrium was reached. In contrast, for most pretreated samples, the *p*-value decreased to the range of 1.1–1.3. A lower *p*-value signifies a flatter extraction curve, where the extraction process begins at a relatively high rate at an earlier time point. Kinetically, this confirms that SE pretreatment mechanically compromises cell integrity. It effectively bypasses the slow “wetting-swelling-cell wall rupture” phase needed for untreated materials. As a result, a substantial amount of polyphenols are released immediately, shifting the kinetic curve leftward and altering its shape. Considering the three parameters A_2_, x_0_, and p collectively, the pretreatment conditions of 1 MPa and 10 min achieved the best balance between high yield (maximum A_2_) and high efficiency (minimum x_0_).

In summary, the extraction kinetics study provides compelling evidence from three key dimensions—rate, efficiency, and final yield—to highlight the superiority of SE pretreatment. The synergistic changes in A_2_, x_0_, and *p* reveal a core mechanism: SE does not merely facilitate diffusion but fundamentally redefines the extraction’s starting point. It transforms the raw material from a dense structure requiring slow wetting and cell wall rupture into a preprocessed, porous matrix perforated with microchannels, thereby enhancing permeability. This structural transformation accounts for the doubled extraction rate (halved x_0_), the altered shape of the extraction curve (decreased *p*-value), and ultimately, more than 60% increase in extraction potential (increased A_2_). Consequently, our analysis validates SE as an innovative strategy that fundamentally enhances the extraction process to achieve high efficiency and high yield simultaneously. These findings establish a solid scientific foundation for the industrial-scale application of SE in extracting plant bioactive compounds.

### 3.6. High-Resolution MS

**Figure 7:**
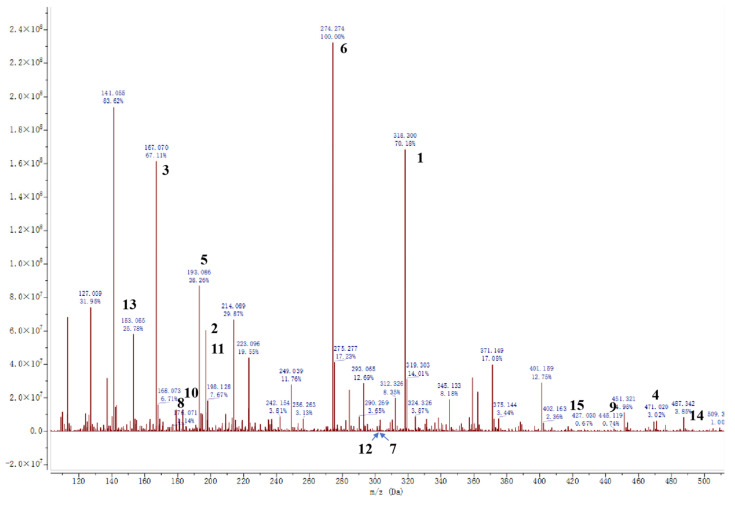
Mass spectrometric characterization of valonea extract by ESI-MS in the positive ion mode. The numbers in the figure correspond to the 15 identified compounds, with detailed information provided in Table 2.

We identified various natural constituents in the valonea extract using high-resolution ESI-MS in positive ion mode. The identification was primarily based on accurate molecular mass, MS/MS fragmentation patterns, and ion abundance ratios, through cross-referencing with the Human Metabolome Database (http://www.hmdb.ca) (accessed on 23 May 2025), PubChem (https://pubchem.ncbi.nlm.nih.gov/) (accessed on 23 May 2025), the mzCloud database (https://www.mzcloud.org) (accessed on 23 May 2025), and relevant literature. The results are presented in Figure 7 and Table 2. The process involved comparing our mass spectrometric data with standard molecular weights, known fragmentation patterns, and published data. The identified natural constituents of valonea extract were primarily phenolic compounds, including six phenolic acids, six polyphenols, one amino acid, and two terpenoids. The molecular weights, classifications, MS fragmentation ions, and molecular formulae of the 15 identified compounds are presented in Table 2.

Compound **1**, exhibiting an [M+H]^+^ ion at *m*/*z* 318.3 and displaying highly characteristic MS^2^ fragment ions at *m*/*z* 147.04, 117.03, and 108.02, along with a relatively high ion abundance indicating substantial content, was identified as loureirin B. The [M+H]^+^ ion for Compound **2** was observed at *m*/*z* 197.1, with MS^2^ fragments at *m*/*z* 109.03, 151.06, and 136.04, corresponding to side chain loss and −CO_2_ + H fragments, respectively. Based on this distinctive pattern and moderate ion abundance, Compound **2** was identified as dihydroferulic acid. The [M+H]^+^ ion at *m*/*z* 167.1 identified Compound **3** as phloretic acid, where the MS^2^ fragments at *m*/*z* 121.06, 107.05, and 149.06 were attributed to ions such as a *p*-hydroxystyrene fragment and a *p*-quinone methide fragment. Compound **4** had an [M+H]^+^ ion at *m*/*z* 471.0 and MS^2^ fragments at *m*/*z* 139.03, 179.03, 247.08, 138.02, and 242.15. The low ion abundance identified Compound **4** as epigallocatechin gallate. The [M+H]^+^ ion at *m*/*z* 193.1 belonged to Compound **5**, which displayed characteristic MS^2^ fragments at *m*/*z* 126.17 and 108.95. This high ion abundance identified Compound **5** as gallic acid. Compound **6** had an [M+H]^+^ ion at *m*/*z* 274.3 and MS^2^ fragments at *m*/*z* 122.06 and 107.07, similar to those of phloretic acid, along with high ion abundance, leading to the identification of Compound **6** as phloretin [36]. Both compounds **7** and **12** had [M+H]^+^ ions at *m*/*z* 303.0. Compound **7**, with MS^2^ fragments at *m*/*z* 259.02, 283.08, and 184.93 and moderate abundance, was identified as ellagic acid. Compound **12**, however, with MS^2^ fragments at *m*/*z* 195.03 and 167.07 was identified as quercetin. The [M+H]^+^ ion at *m*/*z* 183.1 belonged to Compound **8**, which together with MS^2^ fragments at *m*/*z* 178.09 and the decarboxylation fragment 136.95 identified this compound as dihydrocaffeic acid. Notably, the corresponding [M-2+H]^+^ ion for caffeic acid was not detected [37]. Compound **9** displayed an [M+H]^+^ ion at *m*/*z* 443.2 and characteristic MS^2^ fragments at *m*/*z* 179.03, 247.08, 138.02, and 242.15, sharing similarities with fragments of catechin and gallate esters. The low ion abundance supported identification of Compound **9** as catechin gallate. The [M+H]^+^ ion of Compound **10** appeared at *m*/*z* 185.0, with MS^2^ fragments at *m*/*z* 141.19, 126.17, and 108.95 and moderate ion abundance, identifying it as methyl gallate. Compound **11** had an [M+H]^+^ ion at *m*/*z* 197.1 and MS^2^ fragments at *m*/*z* 100.08 and 132.09, characteristic of arginine, leading to its identification as arginine [37]. Compound **13**, protocatechuic acid, exhibited an [M+H]^+^ ion at *m*/*z* 153.0 and an MS^2^ fragment at *m*/*z* 109.02, with low ion abundance. Compounds **14** and **15** were terpenoids. Compound **14** had an [M+H]^+^ ion at *m*/*z* 488.3 and MS^2^ fragments at *m*/*z* 471.34, 423.32, and 287.25, identifying it as ilexolic acid. Compound **15** displayed an [M+H]^+^ ion at *m*/*z* 427.0 and characteristic MS^2^ fragments, including [M-iPr+H]^+^ at *m*/*z* 383.29 and carbon chain loss fragments at *m*/*z* 312.21, 202.10, and 137.06, along with low ion abundance, which supported its identification as stigmast-4-ene-3,6-dione.

### 3.7. Antioxidant Activities of Valonea Extract

**Figure 8:**
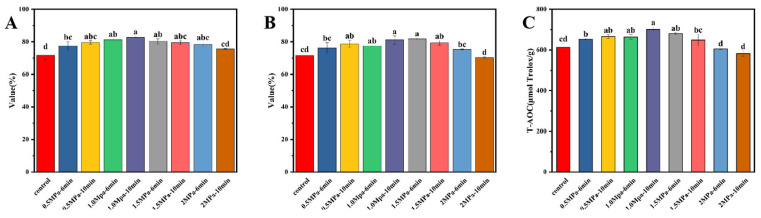
Antioxidant activity of valonea subjected to SE pretreatment under different conditions. (**A**) ABTS, (**B**) DPPH, and (**C**) FRAP. Bars labeled with different letters are significantly different (*p* < 0.05) as determined by one-way ANOVA followed by Tukey’s post hoc test.

The antioxidant activity of valonea extract subjected to SE pretreatment under different conditions was evaluated and analyzed using ABTS, DPPH, and FRAP assays (Figure 8). As pretreatment severity increased, the FRAP activity significantly improved (*p* < 0.05), reaching its peak at 1.0 MPa and 10 min. Beyond this point, further intensification led to a decline in activity. A parallel trend was observed in the ABTS and FRAP assays throughout the pretreatment duration, which was consistent with the change in TP content.

**Figure 9:**
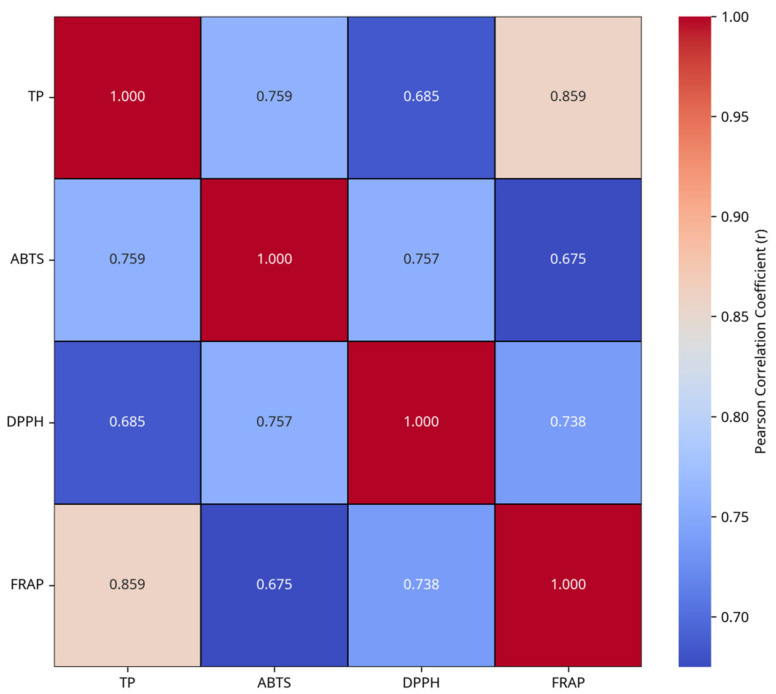
Correlation heatmap between TP content and antioxidant effects.

To quantitatively validate these observations, Pearson correlation analysis was performed. The results revealed highly significant positive correlations (*p* < 0.001) between TP content and all three antioxidant assays (Figure 9). Specifically, TP content exhibited the strongest correlation with FRAP assay (r = 0.8592), followed by ABTS assay (r = 0.7594) and DPPH assay (r = 0.6852). These robust statistical results provide conclusive evidence that changes in the TP content of the extract are significantly correlated with variations in its antioxidant capacity, thereby strongly supporting the initial observation that trends in antioxidative effects are consistent with changes in TP content, consistent with previous studies [13,38,39], which is firmly corroborated by our data. Additionally, our investigation on the quantitative relationships between phenolic composition and antioxidant efficacy confirms the primary role of TP, as demonstrated by its strong correlation with antioxidant activities (DPPH, ABTS, and FRAP). The positive correlation between the concentrations of specific phenolic acids (gallic acid and ellagic acid) and both TP content and antioxidant values suggests that these compounds are likely key contributors among TPs. Therefore, while TP content serves as a robust indicator of antioxidant potential, future research should focus on the bioavailability and synergistic effects of individual phenolic compounds to fully elucidate the underlying antioxidant mechanisms in valonea.

### 3.8. Anti-Inflammatory Activities

Plant polyphenols are widely recognized for their anti-inflammatory properties, suggesting potential applications in inflammatory disease intervention. Valonea extract, a natural source abundant in polyphenols such as ellagic acid and gallic acid, shows significant promise for bioactivity exploration.

**Figure 10:**
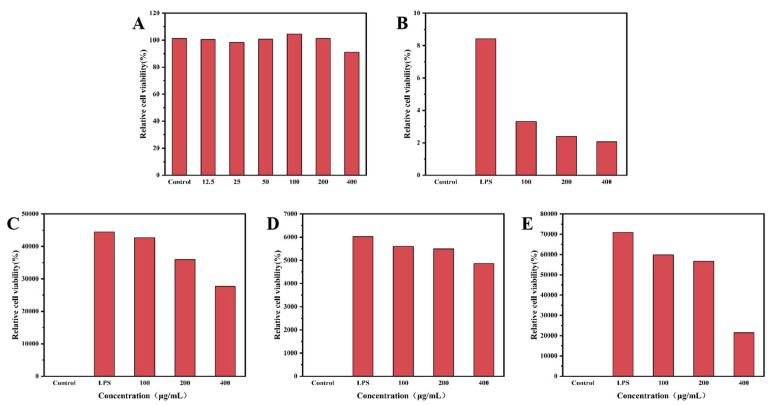
Effects of valonea extract on the viability and LPS-induced inflammatory responses of RAW 264.7 macrophages. (**A**) Cell viability as determined by the CCK-8 assay. (**B**) NO production was measured by the Griess method. (**C**–**E**) Secretion levels of proinflammatory cytokines (**C**) TNF-α, (**D**) IL-6, and (**E**) IL-1β as determined by ELISA.

As shown in Figure 10, LPS stimulation remarkably upregulated NO levels in the supernatant of cultured cells. However, treatment with varying concentrations of valonea polyphenolic extract reversed this effect in a concentration-dependent manner, leading to significant inhibition of NO release. The CCK-8 assay confirmed that the extract did not affect cell viability at any tested concentration, indicating that the observed anti-inflammatory effects were not due to cytotoxicity and supporting the safety profile of the polyphenols.

The cascade amplification of proinflammatory cytokines is the central mechanism regulating the inflammatory response. LPS successfully induced high expression of TNF-α, IL-6, and IL-1β. ELISA results showed that treatment with 100 μg/mL of valonea polyphenolic extract significantly suppressed (*p* < 0.05) the secretion of all three cytokines.

Mechanistically, polyphenols are recognized as effective inhibitors of the nuclear factor (NF)-κB signaling pathway. We speculate that the anti-inflammatory mechanism of valonea polyphenols may involve the following aspects. First, the abundant phenolic hydroxyl groups can effectively scavenge reactive oxygen species (ROS), thereby attenuating ROS-mediated activation of the NF-κB pathway. Second, these groups may directly interfere with LPS binding to Toll-like receptor 4 or inhibit downstream IκB kinase activation, suppressing IκBα phosphorylation and proteolysis, and consequently preventing nuclear translocation of the NF-κB p65 subunit [40,41]. Suppressing this upstream pathway can synchronously downregulate the transcription of inflammatory effector genes, including inducible nitric oxide synthase, TNF-α, IL-6, and IL-1β, which aligns with the multi-cytokine inhibition observed here. Additionally, the potential inhibitory effect of polyphenols on the mitogen-activated protein kinase signaling pathway should not be overlooked [42,43].

In conclusion, these findings demonstrate that valonea polyphenolic extract can safely and effectively inhibit LPS-induced inflammatory responses in macrophages. The fundamental process primarily involves regulation of critical inflammatory cascade components, such as NF-κB, by polyphenolic compounds, leading to suppressed production of NO and multiple proinflammatory cytokines. These results establish a scientific foundation for utilizing valonea extract as a potential natural anti-inflammatory agent.

## 4. Conclusions

The results of this study comprehensively support the core hypothesis that SE pretreatment effectively disrupts the dense lignocellulosic structure of valonea through a synergistic effect of physical forces and chemical reactions, thereby releasing its internal phenolic compounds and enhancing their bioactivity. Research indicates that SE exerts a substantial impact on the material across macroscopic to microscopic scales. Macroscopically, it manifests as significant darkening and a color shift toward reddish-brown hues; microscopically, the disruption of fiber morphology and increased surface porosity, as revealed by SEM, collectively confirm the pronounced effects of SE. The optimal condition of 1.0 MPa for 10 min was identified to maximize phenolic yield, effectively breaking down the lignocellulosic barrier while minimizing destructive side reactions. FTIR and XRD analyses confirmed the hydrolysis of hemicellulose, cleavage of lignin bonds, and reduction in cellulose crystallinity, which collectively enhanced solvent accessibility. Extraction kinetics further revealed that SE fundamentally transformed the material into a pre-fragmented, porous matrix, resulting in a doubled extraction rate and over 60% increase in extraction potential. ESI-MS identified 12 major phenolic constituents, providing a compositional basis for the observed bioactivities. These specifically released phenolic compounds collectively contributed to the remarkable antioxidant and anti-inflammatory activities of the extract, confirming the dual efficacy of SE pretreatment in enhancing both compound yield and functional quality.

In summary, this study demonstrates that SE pretreatment is a highly efficient strategy to enhance the bioactivity and availability of phenolics in valonea. The identified process conditions offer a clear and practical pathway for translating this laboratory success into industrial practice. The efficiency of the method and its alignment with green principles position the resulting extract as a sustainable and potent natural ingredient. Thus, this research provides a compelling blueprint for the industrial valorization of valonea, particularly as a high-value functional additive for animal feed, where its antioxidant properties can promote animal health, or for functional foods and pharmaceuticals, where its anti-inflammatory and antioxidant activities could provide significant health benefits. A limitation of this study is that it was conducted at a laboratory scale, and the bioactivity of the extract was evaluated by in vitro assays. Hence, future research should focus on pilot-scale validation to firmly establish the techno-economic feasibility of SE pretreatment; moreover, in vivo studies should be conducted to confirm the efficacy and safety of the obtained extract in practical applications. Additionally, exploring the applicability of this SE pretreatment to other underutilized agricultural byproducts could further broaden the impact of this technology.

## Figures and Tables

**Table 1:** Impact of SE conditions on the kinetics of phenolic extraction from valonea.

Sample	A_2_ (mg/g)	x_0_ (min)	*p*	R^2^
Control	388.95426 ± 9.43556	11.54059 ± 0.96452	1.77479 ± 0.22152	0.9931
0.5 MPa-6 min	549.08216 ± 9.51691	7.29869 ± 0.43312	1.17193 ± 0.0954	0.99782
0.5 MPa-10 min	567.59228 ± 11.76504	6.83526 ± 0.49375	1.16763 ± 0.11876	0.99676
1 MPa-6 min	509.56125 ± 8.17797	8.41025 ± 0.45568	1.23287 ± 0.08833	0.99809
1 MPa-10 min	633.62246 ± 14.94848	5.77421 ± 0.48996	1.11002 ± 0.13843	0.99594
1.5 MPa-6 min	500.5555 ± 10.09776	8.57882 ± 0.59122	1.27969 ± 0.11773	0.99673
1.5 MPa-10 min	472.94735 ± 7.25013	9.45111 ± 0.4858	1.31104 ± 0.08793	0.99817
2 MPa-6 min	476.36278 ± 5.44033	9.53215 ± 0.35886	1.26801 ± 0.06108	0.99907
2 MPa-10 min	362.19496 ± 5.34553	12.74958 ± 0.60884	1.70708 ± 0.11866	0.99789

**Table 2:** Mass spectrometric data of 15 compounds identified from valonea extract.

No.	Compounds	Molecular Formula	Type	MS/MS (+)	Fragments (+) (*m*/*z*)	Ion Abundance
1	Loureirin B	C_17_H_18_O_5_	Polyphenol	318.3	147.04; 117.03; 108.02	1.5 × 10^10^
2	Dihydroferulic acid	C_10_H_12_O_4_	Polyphenol	197.1	109.03; 151.06; 136.04	5 × 10^9^
3	Phloretic acid	C_9_H_10_O_3_	Polyphenol	167.1	121.06; 107.05; 149.06	1.5 × 10^10^
4	Epigallocatechin gallate (EGCG)	C_22_H_18_O_11_	Polyphenol	471	139.03; 179.03; 247.08; 138.02; 242.15	8 × 10^8^
5	Gallic acid	C_7_H_6_O_5_	Polyphenol	193.1	126.17; 108.95	1.2 × 10^10^
6	Phloretin	C_15_H_14_O_5_	Polyphenol	274.3	122.06; 107.07	1.6 ×10^10^
7	Ellagic acid	C_14_H_6_O_8_	Polyphenol	303	259.02; 283.08; 184.93	8 × 10^8^
8	Dihydrocaffeic acid	C_9_H_10_O_4_	Polyphenol	183.1	178.09; 136.95	1.5 × 10^9^
9	Catechin gallate	C_22_H_18_O_10_	Polyphenol	443.2	179.03; 247.08; 138.02; 242.15	1.5 × 10^8^
10	Methyl gallate	C_8_H_8_O_5_	Polyphenol	185	141.19; 126.17; 108.95	6 × 10^8^
11	Arginine	C_6_H_14_N_4_O_2_	Amino Acid	197.1	100.08; 132.09	9 × 10^9^
12	Quercetin	C_15_H_10_O_7_	Polyphenol	303.1	195.03; 167.07	3 × 10^6^
13	Protocatechuic acid	C_7_H_6_O_4_	Polyphenol	153	109.02	7 × 10^7^
14	Ilexolic acid	C_30_H_48_O_4_	Terpenoids	488.3	471.34; 423.32; 287.25	5 × 10^6^
15	Stigmast-4-ene-3,6-dione	C_29_H_46_O_2_	Terpenoids	427	383.29; 312.21; 202.10; 137.06	2 × 10^6^

## Data Availability

The original contributions presented in the study are included in the article. Further inquiries can be directed to the corresponding author.
